# ‘As soon as they can hold a glass, they begin taking alcohol’: a qualitative study on early childhood substance use in Mbale District, Uganda

**DOI:** 10.1186/s12889-022-13140-w

**Published:** 2022-04-23

**Authors:** V Skylstad, JS Nalugya, AMS Skar, C Opesen, G Ndeezi, ES Okello, KM Moland, IMS Engebretsen, JK Tumwine

**Affiliations:** 1 Centre for International Health, Department of Global Public Health and Primary Care, Faculty of Medicine, University of Bergen, Bergen, Norway; 2 Department of Psychiatry, School of Medicine, Makerere University College of Health Sciences, Kampala, Uganda; 3 Department of Psychiatry, Mulago National Referral and Teaching Hospital, Kampala, Uganda; 4 Norwegian Centre for Violence and Traumatic Stress Studies, Oslo, Norway; 5 Department of Sociology and Anthropology, School of Social Sciences, Makerere University, Kampala, Uganda; 6 Department of Paediatrics and Child Health, Makerere University College of Health Sciences, Kampala, Uganda; 7 Mwanza Intervention Trials Unit, National Institute for Medical Research, Mwanza Campus, Mwanza, Tanzania; 8 Kabale University School of Medicine, Kabale, Uganda

**Keywords:** Early childhood substance use, Social determinants of health, Community child health

## Abstract

**Background:**

Globally, substance use is a leading contributor to the burden of disease among young people, with far reaching social, economic and health effects. Following a finding of harmful alcohol use among 5-8-year-old children in Mbale District, Uganda, this study aims to investigate community members’ views on early childhood substance use among children below the age of 10 years.

**Methods:**

In 2016, we conducted eight focus group discussions with 48 parents and 26 key informant interviews with teachers, health workers, alcohol distributors, traditional healers, religious leaders, community leaders and youth workers. We used thematic content analysis. Four participants and two research assistants reviewed and confirmed the findings.

**Results:**

*Alcohol in everyday life: ‘Even children on laps taste alcohol’:* Almost all participants confirmed the existence of and concern for substance use before age 10. They described a context where substance use was widespread in the community, especially intake of local alcoholic brews. Children would access substances in the home or buy it themselves. Those living in poor neighbourhoods or slums and children of brewers were described as particularly exposed.

*Using substances to cope: ‘We don’t want them to drink’:* Participants explained that some used substances to cope with a lack of food and resources for childcare, as well as traumatic experiences. This made children in deprived families and street-connected children especially vulnerable to substance use. Participants believed this was a result of seeing no alternative solution.

**Conclusions:**

To our knowledge, this is the first study to describe the context and conditions of childhood substance use before age 10 in Mbale District, Uganda. The study shows that community members attributed early childhood substance use to a social context of widespread use in the community, which was exacerbated by conditions of material and emotional deprivation. These social determinants for this practice deserve public health attention and intervention.

## Background

Globally, substance use is one of the leading contributors to the burden of disease among young people [1, 2] and is associated with adverse social and health consequences, including mental illness, infectious diseases, cancer, accidents, and violence [3–5]. Research on early onset of substance use has thus far focused on adolescent years, as this is a period characterised by an increase in risk-taking behaviour, including substance use [1]. Although there is limited data on preadolescent and early childhood substance use, especially from low- and middle-income countries, some reports do exist. Data from the Global School-Based Student Health Survey show that the prevalence of alcohol intake before age 11 ranged from 4.1-43.5% in 45 low- and middle-income countries [6], while in Uganda in 2003, 20.6% of students reported onset of alcohol intake before age 13 [7]. Onset of intake before age 11 has shown a stronger association with adult alcohol dependence, when compared with adolescent onset [8]. Furthermore, early intake of substances can affect the developing brain and key cognitive and emotional functions, such as planning, learning and social development [9, 10].

Children learn from their environment, and early onset of substance use is associated with family and peer practices [11]. Studies have shown that children have knowledge about alcohol from age two, and they understand alcohol related norms and form expectancies from age four [12]. Parental and peer use are important predictors for early use [6, 13, 14], and around age 10 there seems to be a shift from primarily family-oriented to peer-oriented influence [9, 10] and from primarily negative to positive alcohol expectancies [12]. Familial, or household, alcohol supply has been associated with earlier onset of alcohol intake and a higher frequency of drinking [6]. Living with someone with an alcohol use disorder before age 10 has been associated with increased self-reported drunkenness among adolescents in Burkina Faso, Ghana, Uganda, and Malawi [15].

Alcohol consumption patterns vary across the globe. While alcohol per capita consumption is higher in high-income countries and high-income strata of populations, the drinking patterns in low- and lower-middle-income countries are characterised by a combination of high abstention rates in the population and high rates of heavy episodic drinking among those who drink [5]. The Ugandan population has a long-standing practice of alcohol intake [16, 17]. Although 32.5% of men and 62% of women are lifetime abstainers from alcohol [5], Uganda had the world’s highest rate of alcohol consumption in 2004 [18], and the rate of alcohol-use disorder among both male and female adults was almost double the average of the African region in 2016 [5]. A study among secondary school students in central and northern Uganda found that 23.3%, used alcohol, and approximately 10% used kuber (a form of tobacco that can be mixed with other substances), khat, aviation fuel or cannabis [19]. According to the 2018 World Health Organization (WHO) Global Status Report on Alcohol and Health, 86% of all consumption in Uganda was comprised of unregulated local brews, such as fermented beverages made of banana, sorghum, millet, or maize [5]. While it is more acceptable for men to drink publicly [17], brewing is primarily done by women, which has been linked to childhood exposure of brew [17, 20, 21].

In 2014, our team identified an unexpectedly high prevalence of 8.4% clinically defined harmful alcohol use or dependence among 5-8-year-old children in Mbale District, Uganda [22]. While the study sample was small (*n*=119) and consisted of children who had screened positive for a high mental health symptom load, we believe the finding merits further exploration. Substance use in this age group has not, to our knowledge, been the primary subject of investigation in studies from Uganda, while the Ugandan Government’s Ministry of Health’s *Child and Adolescent Mental Health Policy Guidelines* from 2017 state that *“alcohol and drug abuse in children and adolescents in Uganda is on the increase although not well researched”* [23].

In this paper we explore the perception of parents and key informants related to the context and conditions for substance use among children (younger than 10 years) in Mbale District, Uganda. An understanding and appreciation of the social determinants of health underpin our analysis. We discuss our findings considering the WHO conceptual framework for action on the social determinants of health [24]. This framework describes the interplay between structural and intermediary determinants of health, adopting a life-course and socio-ecological perspective. The structural determinants describe the *distribution* of social determinants related to governance, culture, and power dynamics, while the intermediary determinants include material, behavioural, biological, and psychosocial factors, as well as the health system [24]. We use this framework because we believe alcohol and substance use in early childhood is best understood when considering the wider social environment of the child, family, and community, rather than exploring specific pathways for deviant behaviour in children.

The present paper is the first of two papers from this study. While the current paper explores the structural and intermediary determinants related to family and community contexts and conditions for early childhood alcohol and substance use, the second paper explores how this is addressed and managed at a community, institutional (including health system) and government level, and relevant social determinants related to power, social cohesion and agency.

## Methods

### Study design

A qualitative study design was deemed appropriate to investigate thoughts, experiences and practices related to the context and conditions of childhood substance use. We combined key informant interviews (KIIs) and focus group discussions (FGDs), allowing for exploration of agreement and disagreement between participants in groups, while accessing sensitive topics in the confidentiality of individual interviews.

### Study setting

The study was conducted in the Mbale District in eastern Uganda from April-June 2016. Mbale lies by the foot of mount Elgon, approximately 250 kilometres east of the capital, Kampala, close to the border of Kenya and south of the pastoral areas of Kenya, South Sudan, and northern Uganda. Large lines of transportation of goods run through Mbale. It is home to several ethnic groups, including the Bamasaba, Banyole, Bagwere, Baganda, Iteso and Karamojong. The main languages are Lumasaaba and English. According to the latest census of 2014, Uganda had a population of approximately 35 million, with 48% under 15 years [25]. Mbale District had a population of approximately 490 000, with 95,000 living within the urban centre of Mbale City [25]. The census shows that many social indicators are similar to the national average. 13.9% of the children aged 6-12 years were not in school (the national proportion was 12.5%), with rates varying from 8-29% within the Mbale District [25]. 77% percent of the households engaged in either crop growing or livestock farming, a proportion slightly higher than the national average of 69%. 24% had access to electricity, compared to 20% in the rest of the country. The illiteracy rate was 29% among those above 18 years, and 2.9% had education exceeding the secondary level [25]. 9.6% of households consumed less than two meals a day [25]. We anticipate that the situation may have deteriorated for many during the Covid-19 pandemic. There are large slum areas in the district that house approximately 40,000 people, mostly poverty-stricken families, and internally displaced peoples from the formerly war affected north [26]. Most inhabitants in Mbale’s slum areas were renting their housing from landlords and 90% of the inhabitants were low-income earners, with an average daily income of 3000-5000 Ugandan Shillings (0.8-1.3 US dollars) [26].

### Participants and research team

We conducted eight focus group discussions (FGDs) with six participants in each group. We purposively sampled parents of children below 10 years, as they were assumed to have a rich experience with children of the relevant age group. Furthermore, to explore different perspectives, we aimed to include participant groups representing varying characteristics relating to gender, age, and community profiles, i.e., urban/rural residency, slum areas and agricultural areas. To identify participants that suited the purpose of our study and to organise the FGDs, the research assistants collaborated closely with community mobilisers who knew the community well through their experience with community work, for example as head of the women’s committee. The mobilisers were known to the team from previous research projects [27] and had experience with research, recruitment, and ethical procedures, such as confidentiality and voluntary participation. The research assistants carefully explained the purpose of the study and the importance of recruiting focus group participants who had the experience of parenting children below age 10. The participants were recruited into separate discussion groups according to their gender (male or female) and age (18-30 years or 31 years and older), to ensure variability of perceptions between the groups, while preserving homogeneity within the groups. The mobilisers identified and recruited relevant participants directly through their community network and organised a suitable time and place for the FGD in the participants’ home communities. We did not collect information on how many were approached, and how many declined to take part. Before the discussion started, we informed the participants about the study and collected data on age, education level and occupation (Table 1). None of the participants refused to participate or dropped out after inclusion. The FGDs were facilitated by two research assistants with a bachelor's degree in social sciences and community psychology and with experience in qualitative research. Both were fluent in the local language. One of the research assistants moderated the discussion and the other observed and took notes. The first author was not present at the FGDs, since she did not speak the Lumasaaba language, and thus could not take active part in the discussion. We deemed that her passive presence as a foreigner could potentially disturb the discussion. This was based on experience from previous research and advice from Ugandan colleagues.

**Table 1:** Participant characteristics

**Focus group discussion with parents**		**Key informant interviews**	
	*N*		*N*
**Total**	48	**Total**	31
Female	24	Female	14
Younger age (mean: 24 years, range: 18-30)	30		
Older age (mean 49 years, range: 31-76)	18		
*Main occupation*		*Main occupation*	
Farmer	24	Primary school teacher	2
Student	6	Health worker	5
Trader	5	Youth worker	5
Craftsperson	4	Lawyer	1
House wife	2	Police officer	1
Local chairman	2	Mental health activist	2
Qualified professional	2	Religious leader	1
None	1	Alcohol distributor	3
No answer	2	Pharmacist	1
*Education level*		Community stakeholder for children	8
Primary (P1-P7) only	21	Government official	1
Secondary (S1-S6) only	20	Traditional healer	1
High school, A level	1		
Tertiary degree	3		
No formal education	1		
No answer	2		

We recruited 31 participants for 26 key informant interviews (KIIs). We purposively sampled participants we believed had relevant knowledge about childhood substance use, including community leaders, teachers, youth workers, religious leaders, police, health workers, traditional healers, and alcohol distributors (Table 1). We identified participants through our network and by visiting relevant institutions, as well as using snowballing technique, where one participant introduced us to another in person or by phone. The KIIs were mainly individual interviews, while two were group interviews that included three and four participants from the same organisation. Before commencing with data collection, we informed the participants about the study. None refused to participate or dropped out. The first author conducted 23 of the interviews in English, while three KIIs (with a traditional healer and two alcohol distributors) were done by the research assistants in Lumasaaba language. All interviews were done in a location chosen by the participants. At the time of data collection, the first author was a medical student enrolled in a research programme. She had gained experience in qualitative research in the study setting, where she has spent cumulatively one year. At the time of analysis and writing she was a medical doctor enrolled in a PhD-programme.

### Procedures

We used a topic guide for the interviews and discussions. This included topics such as the general use of substances in Uganda, perceptions about childhood use, protective and risk factors, perceived consequences and how they should be handled. The guide was amended during data collection to capture and explore new relevant topics as they arose. To facilitate discussions, the focus groups were read a vignette story about a boy and a girl drinking alcohol before age 10, based on observations in the community. The topic guide was pretested within the research team, and participants confirmed that the story and questions were appropriate and understandable.

Each FGD and KII lasted between 60-120 minutes (average 80 minutes), and were audio recorded. The participants were encouraged to speak openly about their knowledge, opinions, and experiences with the topic. Since the parents and participants had varying levels of education and proficiency in English, the FGDs and three KIIs were held in the Lumasaaba language and were transcribed directly into English by consensus between the research assistants. The remaining KIIs included participants with a certain level of schooling and fluency in English, which is one of the official languages in Uganda. The KIIs were therefore held in English and transcribed verbatim by the first author. The first author and research assistants discussed each transcript during data collection to evaluate the need for further probing, as well as after data collection for clarifications of the content. In the protocol we had estimated a need for 15-30 KIIs and 4-8 FGDs with 4-6 participants in each group. We continued data collection until saturation was deemed met, i.e., when no new themes seemed to arise, and a sufficient variety of key informants was represented.

### Analysis and interpretation

The dataset comprised the transcripts of the KIIs and the FGDs. These were thoroughly read and reread to gain a sense of the whole before, during and after undergoing thematic content analysis [28]. We analysed the FGDs and KIIs as one dataset, as our intention was not to compare them, but rather triangulate methods and populations for comprehensiveness. The first author used NVIVO 12 for open coding of the transcripts, before the team sorted them into categories, sub-themes and themes using Office Word. We used an inductive approach, staying close to the original data in reporting and interpretation of findings. The identified codes and themes were discussed by the team and refined throughout the process of analysis and writing. Quotes were chosen according to their ability to illustrate the essence of the theme. We aimed for a comprehensive and nuanced description of the findings in the social context of Mbale, with a broad representation of perspectives from a varied group of participants. When quotes included more than one participant, they were assigned a number, ‘P1’, ‘P2’, and the interviewer was marked ‘I’. Further, the findings have been complemented by observations made by the first author, including from the media and social media outlets. To provide context to the quotes, we have labelled them with the role for which the participants were purposively sampled but have sought to generalise these terms to ensure the anonymity of the participants.

A draft of the results was shared twice with participants from the KIIs that had consented to be contacted for clarifications later in the process of analysis. Eight participants were invited to provide feedback, and four participants answered and accepted the invitation. The two research assistants provided feedback on behalf of the FGDs. All agreed that the findings reflected a true and accurate representation of their reports and perceptions. None wished to make any changes.

## Results

We identified two main themes related to the context and conditions for early childhood substance use. The first theme *‘Alcohol in everyday life’*, described a context with widespread substance use and brewing for everyday life and traditional celebrations. The second theme *‘Using substances to cope’* described conditions, such as deprivation and traumatic experiences, that exacerbated the substance use by some. The term ‘substance’ has been used to include any psychoactive substance, including alcohol. When appropriate, the substance has been named. While acknowledging the ongoing discussion of the appropriateness of the term ‘slum’ [29], we chose to use this term in addition to the term ‘poor neighbourhoods’, since it was used in the context and a consensus on an alternative term that sufficiently covers the characteristics of slums has not yet been reached.

### Alcohol in everyday life: ‘Even children on laps taste alcohol’

Almost all participants agreed that the problem description and vignette story was recognisable, expressing a consensus on the existence of and concern for childhood substance use before age 10. The participants emphasised a higher frequency of alcohol consumption among adults and teenagers, and the reported amount and frequency of use by children varied. Guesstimates ranged from 10-98% of children drinking, and amounts varied from tasting to a more extensive use from a very young age*: “P: Alcohol, like local brew, you can find even a child of 3 years drinking local brew. (I: 3 years?) P: Yes, three years and he is drinking local brew and holding a cup like an adult” (FGD 7, younger women)*. The participants did not think that it was acceptable for children to drink, but considered the practice to span regions, place of residence, socioeconomic position, and age groups. They explained that the cultural background of the family, living conditions and practices within the home could make some children more vulnerable than others. Some believed that religious conviction could be protective, while others did not perceive it to be important. We note that in one FGD with young women, it was discussed whether substance use happened among children, and one KII participant was hesitant about the importance of the problem, stating that: *“If it is there, it is an ignorable percentage” (KII 16, government official).*

#### ‘Drinking is a part of the culture’

The participants described that alcohol was part of longstanding traditions in the country and intake was widespread. They explained that traditional home brews called ‘malwa’ and ‘waragi’, were most used by the community members, including children. Since malwa is made of grains used for food, some community members claimed that it could not be harmful: *“Malwa is [made of] maize and yeast, and millet. They fry and then add water, after three days it ferments, that is the only difference. So, the parents tell you that, that is food, in the form of liquid, so what is wrong with that? (I: so that it is alcoholic doesn’t really matter) Yes, that it is alcoholic is not bad.” (KII 3, community stakeholder for children).* A small minority of participants claimed that children only drink malwa on day one or two of the fermentation process, while it is not yet alcoholic, but other participants dismissed this. ‘Waragi’ is a distilled liquor, mostly used by adults, but also by some children. A few participants explained that some community members believed that waragi and malwa could treat ailments and make someone sharp and strong: *"There are good things, because when a person drinks, he can go to the field and run for a long time" (FGD 8, younger men).* Further, they shared that some young children used marijuana, which was sometimes grown at home for hens to reduce inflammation and improve appetite. Substances such as kath and kuber (a form of tobacco that could be mixed with other substances), were widespread, while solvents, such as sniffing of glue and fuel, were mostly used by street-connected children.

Alcohol was expected in social gatherings and events but was also integrated in daily life to the extent that people no longer noticed it*: “Drinking is a part of the culture for Mbale and it runs in the family [...] They have their local brew and even gives to a young child. From 5 years, a child knows alcohol. [..] Alcohol here in Uganda… people take it as water" (KII 6, traditional healer)*. Children were especially exposed when alcohol was part of celebrations and ceremonies. Several participants reported a tradition of giving brew to a newborn within the first week of life, to connect with the ancestors. Further, the season of circumcision ceremonies for adolescent boys in Mbale District included especially high intake of and access to alcohol, also for young children. *“In circumcision ceremonies, some families gather the children and tell them to drink local brew to fulfil the celebration of the culture. Even the children who are still on laps are made to taste alcohol. The culture of the bagishu [ethnic group of the Mbale area] brings alcohol for the young children to drink, because all of them are given local brew” (FGD 2, older women).*

Although participants reported that drinking took place in all of Uganda, they emphasised that the north eastern populations, the Karamojong and Iteso, were especially known for their strong culture for brewing and sharing this with their children. Participants from the north eastern area confirmed this notion: *“P: Especially I can speak about where I come from, Karamoja. There I would say children start drinking from day one of their birth, because as soon as you’re born they make sure that you taste the alcohol […] (I: and when would they start sipping, or drinking without their parents minding?) P: As soon as they can hold a glass, they begin taking alcohol.” (KII 10, religious leader).*

#### ‘If the parent is drinking, they also give the child’

There was agreement that children would mainly access substances in the home or buy it themselves. The participants believed that growing up in an environment where parents and older children use substances was an important risk factor for own use. They were concerned that children copied the behaviour of their parents and peers, or were given alcohol by them directly: “*P: The moment the child starts walking it begins to ask for things and so if the parent is drinking, they also give the child (I: At what age?) P: Like one year. (I: And if the child finds you drinking, you give to taste?) P: Yes, I stopped drinking, but when I used to drink, I would also give my child to drink some, which was really bad.” – (FGD 2, older women)*. The participants explained that children are allowed, and often asked, to buy alcohol on behalf of adults, and they believed many children started tasting in this process. Peer influence was also considered important, especially in cinema halls and in school: *“Almost all schools have bars around. The child goes to school to study, but at break time the children go to sit in those bars and the bar owners do not care to say anything because they are also looking for money” (FGD 8, younger men).* Sales of alcohol to children was observed first-hand when an eight-year-old child bought a small bag of liquor during an interview with a bar owner.

#### ‘As we brew, children start tasting’

In the homes of brewers, it was considered almost inevitable that children were drinking. One participant, who grew up with parents that brewed, explained: *“Children drink because of the family background. Like in the family I grew up, our fathers and mothers used to cook waragi and therefore there was no way you would skip taking that waragi and the malwa.” (FGD 8, younger men).* A brewer, who was also a parent, confirmed that the appropriate age to start drinking brew was blurred: *“It is hard because we brew from home, and as we brew, children start tasting. So, it is hard to say that at this age it is ok, since we brew it at home.”* (*KII 9, alcohol distributor).* In addition to high availability, the children were also exposed to brew when helping in sales, which included tasting to prove the safety of the brew: *“You put the malwa in the pot, and then you pour hot water. As the seller puts the straw inside, they are told to sip first. When the child has been tasked to serve, they have to first of all sip to see if it poisoned and if the straw functions well and is not blocked. In that process a child gets addicted to the alcohol.” (KII 23, primary school teacher).*

According to the participants, brewing was an important source of income, especially in poor neighbourhoods. The slum areas in Mbale City were described as crowded, with a high density of bars and brewing spots, and were inhabited by mostly low-income families and internally displaced peoples from the north east. To open a bar or brewing spot was considered an attainable source of income in poor neighbourhoods since regulations were poor and demand was high: *“In poor families they will brew local brew to get some money for buying clothes, something to eat and may be buy some flour” (FGD 7, younger women).* The participants described a form of double dependency on the brew, where it was necessary for income, but also made the brewers and their family more exposed to alcohol due to increased access to and use of brew: “*They use substances because of the parent’s situation not being good and they sell alcohol. Whether a Karamojong, or a Gishu, or Mugwere, as longs as he sells alcohol, even the child at home has to drink. Mother drinks, father drinks alcohol even the child has to take alcohol whether of 1 year, or 3 years.” (FGD 1, older men).*

### Using substances to cope: ‘We don’t want them to drink’

The participants explained that rich, poor, urban, rural, educated, and uneducated people used alcohol and substances, and that one such factor alone did not explain why children were exposed to this. Rather, they explained that growing up with a set of poor social conditions exacerbated the existing practice of intake. While the participants believed that some parents were unaware or did not care about the harm of substance use, and that some children should be disciplined for experimenting, they explained that many had no choice. They described that a complex interplay between social, economic, and cultural circumstances resulted in childhood exposure of substances as part of coping with deprivation and psychological stress. In these cases, despite knowing the harmful effects of substance use, the parents did not have the resources to protect their children from it.

#### *‘They call it ‘My food, my blanket’’*

The participants explained that in some families the local brew was used to alleviate hunger and help the children sleep: *“They drink because of hunger and [for the children] to not disturb you because there is nothing [to offer them]. Every child drinks, and they end up sleeping. [..] It is to cool the hunger. We don’t want them to drink… We know that it’s bad, but a kilo of posho [staple food] cost a lot of money and if you have 11 children, how will you feed them? You buy a jerry can of local brew and put it there for the children to drink as you are looking for what to eat.” (FGD 8, younger men)*. Alcohol and substances were also used to cope with a lack of capacity and social support for childcare. Although the practice was unknown by some, multiple participants shared experiences with parents giving alcohol or benzodiazepines to children when they did not have the capacity to attend to them: “*The mother of the child gets the alcohol and puts it in the mouth and then gives the young child, but does not give much. Have you ever seen how the doves feed their young ones? Then she sleeps. As you know the baby’s brain is still weak, she sleeps[...] You know how children cry, they disturb the mother so she has to give [alcohol] so that they can sleep and give the mother opportunity to do other things like brewing local brew.” (KII 11, alcohol distributor)*. In some rare cases, this practice also occurred in situations where single parents who worked evenings had to leave the children unsupervised and saw no other choice than making sure the child slept: *“One time there was a lady whom they brought for child neglect, complaining that she goes out in the night and she leaves the children of 4 and 6 years at home. She gives them alcohol so that they sleep […] I have also heard about a nursery school where they would give to children some Diazepam (benzodiazepine), and they will sleep.” (KII 20, health worker).* The participants explained that this practice was not accepted by the community, but they understood it as an act of desperation by caretakers living on the margins.

Street-connected children were also reported to use substances to cope with unmet needs. Substances helped reduce hunger, ease sleep, feel warmth, get courage to beg for money and sleep outside at night and look for food in the rubbish. The main substance used was aviation fuel as it was cheap and could last for a long time if put on a cloth and kept in a plastic bottle. One participant that worked closely with street-connected children explained that ‘jet fuel’ was preferred as it provided favourable effects, and they could not see any other solution: *“You see the kids, they use what is called jet fuel. They call it 'my food, my blanket' because you don't feel hungry and you don't feel cold in the night. Now, why would you want to take away somebody's food and somebody's blanket if you are not providing another solution?” (KII 25, youth worker).*

#### *‘Now it is about forgetting’*

Substances were also used to cope with traumatic experiences and psychological stress. The participants explained that some children who experienced neglect and domestic violence, particularly from stepparents, ran away from home to escape an intolerable situation: *“Some children tell me they run away from home because of abuse. Parents were beating them and they say they can't take it anymore. Then we have kids from families that have a step mother, and they can't live together, and maybe she is denying them food and they decided to run away.[…]. [In the street] they have really hardened. They can't go [from the street], they will die there. But using substances helps them cope with the conditions.” (KII 17, police officer).*

The use of substances to cope with trauma also applied to the north eastern populations whose long-standing traditions for substance use were exacerbated by their experience with war, insurgency, and internal displacement. One participant from the area explained: *“P: After the insurgency, some people had this feeling of dejection and loss. So, they went into the habit of drinking. Drinking has always been a culture in Teso [north eastern area], and local brew is always part of a good welcome home. But after the insurgency, it just went pooff, I think.. [..] it is more for forgetting problems and people were poor and started drinking instead of working. The social drinking was usually in the evening after they have done their work. They sit together and discuss over a pot, but now it is about forgetting.” (KII 20, health worker).* The celebratory and social aspect of using substances had turned into a way of coping with a hopeless situation.

## Discussion

In this study, we explored the context and conditions for childhood substance use before age 10 in Mbale District, as perceived by community members and key informants. Our study shows that widespread use of alcohol and substances as part of the everyday life and local traditions left children exposed to substance use. For some, this was exacerbated by conditions where substances were used to cope with resource deprivation and psychological stress. In the following sections, we will discuss these findings considering the WHO Commission on Social Determinants of Health (CSDH) Framework [24] and existing literature, as we believe social determinants are relevant for all children exposed to alcohol and substance use, yet more specific theories on child behaviour can supplement the insights drawn from the social determinants of health framework.

### The context - structural determinants of health inequality

According to the CSDH framework, the structural determinants of health consider the *distribution* of exposure and vulnerability to health compromising factors, and are twofold. One part includes the socioeconomic and political context, including policy, culture, and societal values, and the second part includes socioeconomic position on three levels: individual, household, and neighbourhood. Socioeconomic position is in broad terms related to social class, gender, ethnicity, income, education, and occupation. In the case of children, these factors are understood as ‘received’ from the parent [24].

The participants explained that children learned from their environment, where substances, especially alcoholic brew, was an important part of daily life, as well as during ceremonies and celebrations. This tended to normalise the use of substances also by children, even if the use in this age group was not fully approved of. Globally, psychoactive substances are part of cultural practices, religious rites, building social bonds and coping with hardship [30]. In Uganda, brewing has been an important part of community life, and has been described as ‘integrated’ into the community [31]. Studies have shown that the intake of alcohol in the north eastern areas was double that of the central region among adults [17] and youth [19]. This is in line with our findings describing a higher vulnerability for alcohol exposure among children from these areas. However, the practice of intake by children of brewers has been described for populations both in the north [21, 32] and in the south [20], suggesting that this may apply to a large part of the country.

The participants explained that while the intake of substances was generally high, it was higher in poor neighbourhoods and slums, where brewing spots were common. In 2010, ACTogether Uganda, Uganda Slum Dwellers Federation and the Municipality of Mbale undertook a profiling of Mbale City and its informal settlements [26]. They described that brewing was a main income generating activity in the slums, and noted that, although there was varying access to social and religious meeting places, *“there are other social places like bars”* [26]. Globally, there have been contradicting results on whether neighbourhood density of alcohol outlets make the populations more vulnerable for alcohol intake. One systematic review found an association [33], while another systematic review was inconclusive, but reported a possible association among adolescents [34]. When interpreting these results, one needs to have context-specific factors in mind. One example of this we found in a study from South Africa where the authors found an association between problem drinking and alcohol outlet density, but not with heavy drinking [35]. They stipulated that a potential explanation for the missing association with heavy drinking was a weekly traditional event with dancing, where home brewed alcohol was sold, providing a source of alcohol consumption that was missed when only focusing on alcohol outlet density [35].

### The conditions - intermediary determinants of health

While the structural determinants of health describe the *distribution* of exposure to determinants, the intermediary determinants describe the relevant interrelated conditions, material circumstances, psychosocial factors, behavioural and biological factors. Social cohesion and networks are placed as a factor overlapping the structural and intermediary determinants. As mentioned, the CSDH adopts a socio-ecological and life-course perspective, and children’s determinants are defined by the conditions of the family [24].

Our findings strongly suggest an important connection between scarcity and increased vulnerability to substance use among children. The participants explained that since substances were so accessible, and by some deemed a nutritious food replacement, they provided an immediate solution to a range of daily life challenges when alternative solutions were not available. Brew and substances were used to generate income, relieve hunger, and help sleep. In lieu of a social network for childcare support, some caretakers used substances to release capacity for work and chores. Street-connected children used substances to mitigate the lack of safe housing, food, and warm clothes. Similar practices for using substances to cope with needs have been described elsewhere. Brewing alcohol has been identified as an important income generating activity among women [17], slum dwellers [26] and displaced populations in Kenya and Uganda [21, 32]. In Karamoja, mothers have reported giving brew to babies and children to help them sleep and relieve hunger [21], while in Pakistan [32] and Nigeria [36] reports show that opium and brew have been used to keep the child calm while the caregiver attends to other chores. These practices do not necessarily imply that caregivers are unaware of the potential harm. In a study from Peru, where 61% of parents reported that their 5–12-year-old children drank brew, with a median 3-year age of initiation, a majority of the parents believed that alcohol may be harmful, but also nutritious with the potential to aid growth at a low price [37]. These practices can be understood in light of Mullainathan and Shafir’s work on how scarcity affects decision-making [38, 39]. The authors highlight that the ‘mental bandwidth’, or capacity, required for good decision-making, may not be available when resources are so scarce and basic needs like food and sleep must be addressed. In this situation, the ‘bandwidth’ for parenting is not available, and people tend to ‘tunnel’ on solving the acute issue at hand, such as income, food, and chores, disregarding long-term outcomes [39].

The participants explained that substances were also used to cope with a lack of security, nurturing care, and processing of traumas. This was especially relevant for two groups, namely the north eastern populations who were victims of war and insurgency, believed to have exacerbated an already strong habit for drinking alcohol, and street-connected children, victims of domestic abuse and neglect, especially by stepmothers. The practice of using substances to cope with psychological stress is well known in the addiction field as ‘self-medication’ [40, 41]. In line with our findings, this association has been documented qualitatively [32] and quantitatively [42, 43] among internally displaced peoples in Uganda, as well as among survivors of childhood trauma [44, 45]. A systematic review on the prevalence of substance use by street-connected children in resource constrained settings found that the prevalence ranged from 15-92%, with variation according to geographical location and methodology [46]. In a survey and qualitative assessment of street-connected children in Kenya, the authors identified ‘peer influence and social network’, ‘coping and survival on the streets’, ‘availability and affordability of drugs’, ‘poverty’ and ‘negative family influence’ as the most important barriers to quitting drugs. Further, 71.4% strongly agreed with the statement ‘Glue helps me cope with reality’ [47].

### Implications for policy

The United Nation’s Sustainable Development Goal 3.5 recognises the need to *“Strengthen the prevention and treatment of substance abuse, including narcotic drug abuse and harmful use of alcohol*” [48]. The CSDH framework provides guidance on policy, calling for context-specific strategies that address both structural and intermediate determinants of health with a multisectoral approach, including community participation [24].

In our findings we observe an interplay between the structural and intermediary determinants, the context that permits childhood substance use and the conditions that exacerbate it. On a structural level, we identify a need for policies that protect children from access to alcohol and substances, and improved opportunities for alternative work and income generation, especially for women, internally displaced peoples, and slum dwelling populations. On an intermediary level it is imperative to address the conditions that make these children and caregivers resort to substances as a coping mechanism, seeing no alternative. While we agree when the CSDH framework states that *‘interventions addressing intermediary determinants can improve average health indicators while leaving health inequalities unchanged’* [24], we acknowledge the acute character of the scarcities described in our findings and believe action targeting the intermediary determinants are necessary on humanitarian grounds. It is crucial to alleviate the described need for food, network, and prevention as well as treatment of traumatic experiences with multisectoral and targeted interventions, while also improving the *"the circumstances in which people grow, live, work, and age*"[49].

### Strengths and limitations

This study provides important and novel knowledge about the context and conditions in which early childhood substance use occurs in Mbale District, Uganda. We included a relatively large sample of participants, triangulated KIIs and FGDs, allowing for comprehensive exploration, and sought participant validation of the findings. Yet, the study has some noteworthy limitations. Firstly, most of the participants reported experiences and observations about other community members, rather than about themselves. Whether this reflects the experiences of the families they describe is therefore uncertain. Some, however, did report from their own lived experience with childhood substance use, verifying the reports. Secondly, we did not conduct interviews with children, leaving factors less known by parents and other adults unexplored. Thirdly, we investigated childhood substance use with a problem focus, missing important aspects such as protective environments, preventative measures, resilience and modifying factors. We acknowledge the value of a more solution-oriented approach of investigating children who grow up in similar contexts and conditions, without developing harmful substance use.

Further, we acknowledge the limitation in using translated transcripts, compromising original expression of concepts [28]. To mitigate this, we had bilingual research assistants who were native speakers of both Lumaasaba and English. The research assistants reached consensus on the translation and were involved in discussing and clarifying the content of the translated transcripts. In addition, since the first author was a foreigner and did not know the Lumasaaba language, she was only present in the KIIs, where she could have an active role and we considered her background would have the least impact on the interview setting and answers. Appreciating her position as an outsider, she discussed the first impressions of the findings with a Ugandan medical anthropologist (ESO) affiliated with the Department of Psychiatry at Makerere University at the time of the study. It is a noteworthy and reassuring observation that the findings in the FGDs and KIIs overlapped to a large degree. The FGDs had only Ugandans present, and their open sharing about this sensitive topic gives us an indication that they were not hampered by a wish to give socially desirable answers in the group setting. Further, a subset of participants gave feedback on the findings after analysis, emphasizing their agreement with the presented themes. Moreover, it is worth noting that the data was collected in 2016 and analysed from 2019-2021 due to capacity issues. The time passed may have left relevant changes unaccounted for, including a relatively recent ban on small bags of hard liquor [50], which are now substituted with small bottles. It is also worth mentioning the harsh consequences of the Covid-19 pandemic, which has led to an exacerbation of the described social conditions for many people, with a potential increase in exposure to alcohol for children. Apart from this, we have not observed other substantial societal changes in Uganda in the past five years that we believe would have large implications for our findings or conclusions.

## Conclusion

In this paper we present findings related to the context and conditions in which children use substances before age 10 in Mbale District, Uganda. Substance use in early childhood was a concern for parents and key informants. Culture and context combined with an acute scarcity of resources, security and care left children exposed to alcohol and other substances, with potentially detrimental effects on public health and opportunities to thrive. We found that living in communities and families with high intake promoted early use, especially in slum areas or in families that brew. Substances were used to cope with deprivation and psychological stress, particularly in situations with lack of food and childcare capacity, as well as traumatic experiences from war or domestic abuse. We have explored and discussed the findings considering the interplay between structural and intermediary social determinants of health described in the CSDH framework, opening an opportunity for meaningful prevention and intervention initiatives targeting these determinants. Real alternatives for income generating activities, hunger relief and trauma processing must be made available. Exploring the children’s own perspectives is warranted, as well as epidemiological investigations of the prevalence, associated risk and protective factors, and long-term consequences for the developing child. Despite the importance of future research, the presented report should be sufficient to entice urgent attention and action targeting substance use in this age group.

## Data Availability

The University of Bergen and Makerere University have shared intellectual property rights to the data. The datasets used and analysed during the current study are available from the corresponding author on reasonable request.

## Abbreviations

FGD: Focus group discussion
KII: Key informant interview
WHO: World Health Organization
CSDH: Commission on Social Determinants of Health
